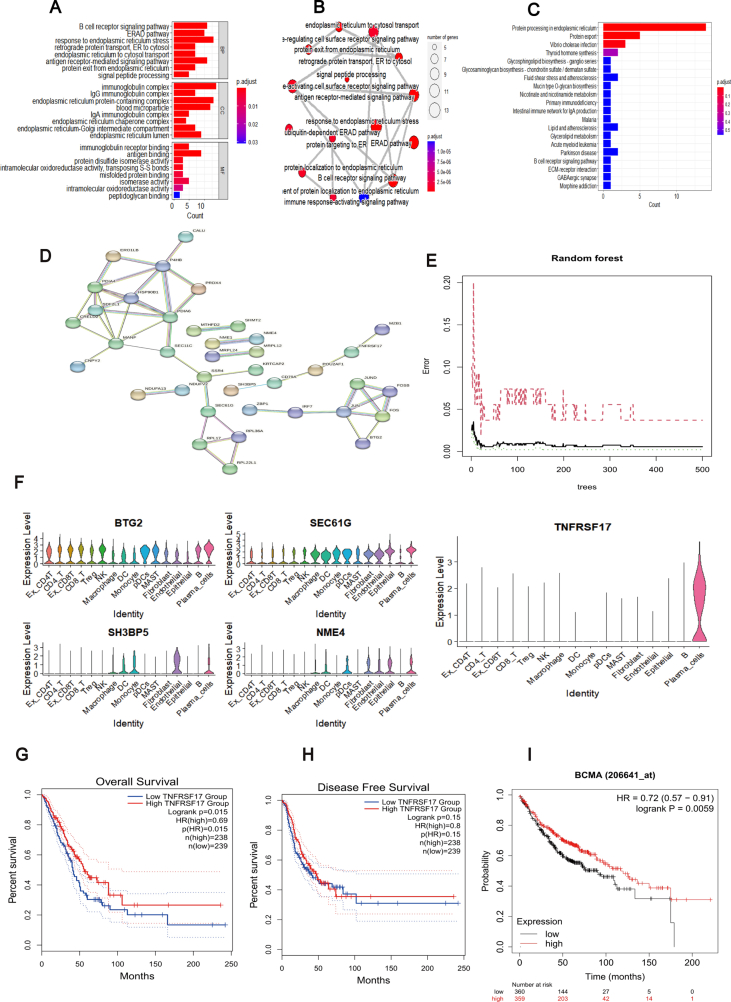# The important role and core marker gene of tumor-infiltrating plasma cells in the microenvironment of lung adenocarcinoma

**DOI:** 10.1016/j.gendis.2024.101274

**Published:** 2024-03-22

**Authors:** Jianhong Zhang, Chengyang Song, Xiuqin Feng, Qian Yu, Xueying Yang

**Affiliations:** Department of Thoracic and Cardiac Surgery, The Fourth Affiliated Hospital of China Medical University, Shenyang, Liaoning 110032, China

Lung adenocarcinoma (LUAD) is the primary subtype of lung cancer, but its prognosis remains challenging. The tumor microenvironment plays a central role in the initiation and development of tumors, particularly regarding the composition of cell types. Understanding the cellular composition within tumors is crucial for comprehending disease processes and improving treatments. Single-cell RNA sequencing (scRNA-seq) provides high-resolution gene expression data for single cells within tumors, allowing for precise analysis of different cell types and their gene expression profiles, but it lacks prognostic information. In contrast, bulk RNA sequencing (bulk RNA-seq) data contains plenty of prognostic information. Therefore, the deconvolution of scRNA-seq combined with bulk RNA-seq containing prognostic information is a favorable approach. In this study, we discovered a positive correlation between a high proportion of tumor-infiltrating plasma cells (PCs) and improved overall survival in LUAD. Through bioinformatics analysis, we identified TNFRSF17 as a potential key factor to improve prognosis in LUAD. This discovery was validated through immunohistochemical staining of clinical samples, suggesting that TNFRSF17 plays a pivotal role in the underlying mechanisms affecting LUAD prognosis.

ScRNA-seq data from eight LUAD of two datasets were obtained and a standard processing procedure was used, which eventually yielded 16 cell types (Fig. 1A). Annotation was performed through combinations of SingleR and marker gene-based manual (Table S1). The expression of marker genes was visualized using stacked violin plots (Fig. 1B). More details on the annotation of scRNA-seq data can be found in File S1 and Fig. S1. Then, we used “BisqueRNA” (version 1.0.5) to deconvolute the TCGA LUAD bulk RNA-seq data based on scRNA-seq to calculate the proportion of 16 cell types for each sample.^1^ Table S2 presents the proportions of these 16 cell types across 503 samples. Next, we calculated various risk factors associated with tumor prognosis (Table S3) and visualized hazard ratios (Fig. 1C). The results revealed that PCs serve as a protective factor, while monocytes, fibroblasts, EX_CD4T cells, and EX_CD8T cells were associated with increased risk in LUAD.

**Figure 1:**
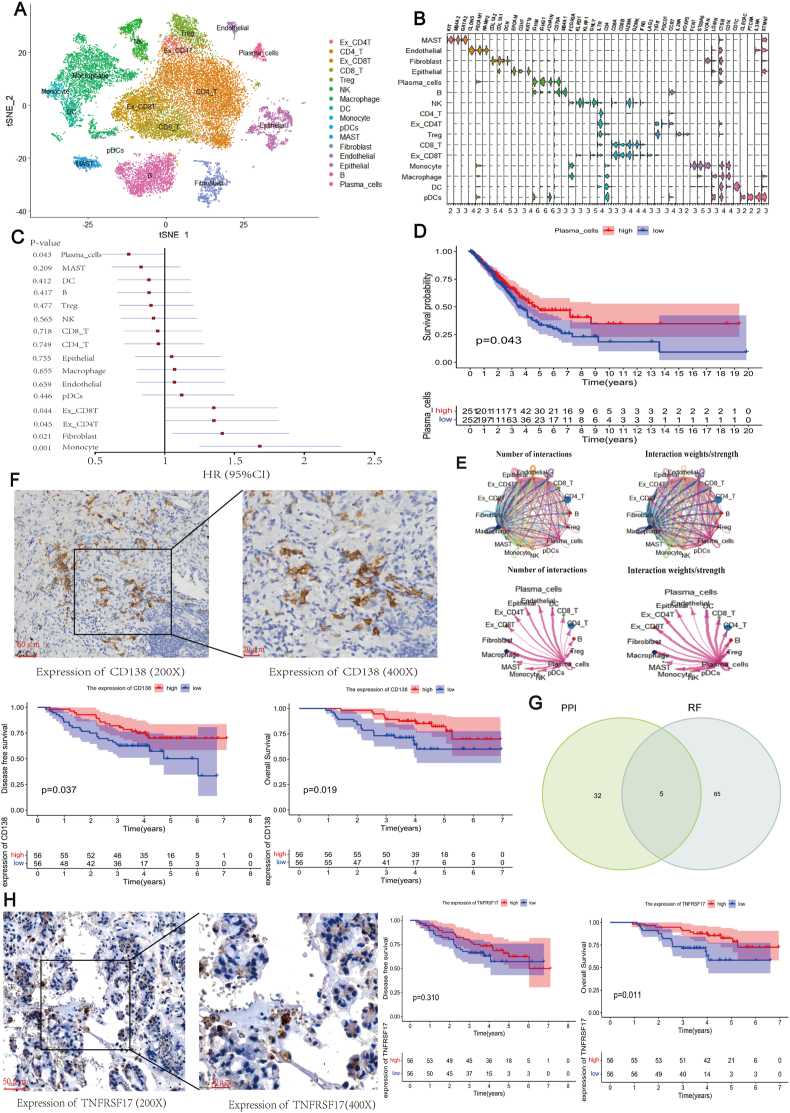
The important role and core marker gene of tumor-infiltrating PCs in the microenvironment of lung adenocarcinoma (LUAD). **(A)** The 29 cell clusters were finally annotated into 16 cell types. **(B)** Expression of marker genes in each cell type. **(C)** Hazard ratios calculated for each cell type based on the deconvolution results. **(D)** Based on the results of the deconvolution of single-cell RNA, 503 LUAD patients were divided into high and low groups according to the number of PCs, and the results showed an association between infiltration of PCs and better overall survival (OS) in LUAD patients (*P* = 0.043). **(E)** Strength and number of interactions of various cell types in the LUAD tumor microenvironment, and strength and number of interactions of PCs with various cell types in the LUAD tumor microenvironment (thick and thin lines represent the strength of the interaction). **(F)** Immunohistochemical (IHC) staining of CD138 was performed on 112 LUAD specimens from our cohort, which were divided into high and low groups according to the degree of infiltration of CD138, and the results showed that LUAD patients with a higher degree of infiltration of PCs had a longer disease-free survival (DFS) (*P* = 0.037) and a better OS (*P* = 0.019). **(G)** Differentially expressed genes were intersected with prognostic genes to obtain the core genes of tumor-infiltrating PCs. **(H)** IHC staining of TNFRSF17 was performed on 112 LUAD specimens from our cohort, which were divided into high and low groups according to the degree of infiltration of TNFRSF17, and the results showed that TNFRSF17 was not significantly associated with DFS of the patients (*P* = 0.31). TNFRSF17 was associated with better overall survival of the LUAD patients (*P* = 0.011).

We further investigated the association between PC infiltration and LUAD survival. Kaplan–Meier curve analysis demonstrated that higher PC infiltration correlated with better survival in LUAD based on deconvolution results (Fig. 1D). Additionally, we assessed the clinical relevance of CD138, a common marker for assessing PC infiltration using TCGA data. High CD138 expression was significantly associated with the early pathological stage and exhibited a tendency to be related to better overall survival (Table S4).

To gain a deeper understanding of the function of PC marker genes in the tumor microenvironment, the study conducted GO enrichment analysis, which revealed a close association of PC marker genes with B cell activation, regulation, and lymphatic system activation (Figs. S2A and B). KEGG enrichment analysis also revealed a strong link between these genes and pathways such as protein processing in the endoplasmic reticulum (Fig. S2C). Using CellChat, complex intercellular communication networks were depicted, showing that interactions between PCs and other immune cells were highly active, while the interactions of PCs with stromal cells were less pronounced (Fig. 1E).

To further validate the role of PCs in LUAD prognosis, we included a cohort of 112 LUAD patients with follow-up data. Immunohistochemical staining was performed on pathological specimens to assess CD138 expression (Fig. 1F), which indicated a correlation between CD138 expression and early clinical pathological stage (Table S5), and patients in the high tumor-infiltrating PC group had a better disease-free survival (*P* = 0.037) and overall survival (*P* = 0.019) (Fig. 1F).

Next, we performed gene-centric differential expression analysis and survival analysis to identify potential marker genes of PCs. A total of 135 differentially expressed genes were obtained by filtering for |log foldchange| >1 and false discovery rate <0.05. We selected validated protein–protein interactions with an integrated score >0.8, resulting in 37 core differentially expressed genes (Fig. S2D). Next, 117 prognosis-associated genes were identified from 942 marker genes by univariate Cox regression. We employed a random survival forest for feature selection with relative importance greater than 0.4, resulting in 70 prognosis-associated genes (Fig. S2E).

We identified 37 core differentially expressed genes and 70 prognosis-related genes from 942 PC marker genes, with an intersection set revealing five core genes: BTG2, SEC61G, SH3BP5, TNFRSF17, and NME4 (Fig. 1G). Among these, TNFRSF17 exhibited specific expression in tumor-infiltrating PCs (Fig. S2F). Notably, TNFRSF17 was also identified as a marker gene specifically expressed in PCs.^2^ We analyzed the clinical relevance of TNFRSF17 and found that TNFRSF17 was significantly associated with old age and early pathological stage in LUAD based on TCGA data (Table S6). Moreover, we observed a significant correlation between TNFRSF17 and better overall survival through the online Gepia database analysis (Fig. S2G) (*P* = 0.015), although disease-free survival was not statistically significant (Fig. S2H) (*P* = 0.15).

Subsequently, we performed additional analysis of TNFRSF17 using the Kaplan–Meier plotter database. The results also confirmed a significant association between TNFRSF17 and improved survival rates in LUAD (Fig. S2I) (*P* = 0.0059). Consequently, we identified the TNFRSF17 gene as a core prognostic-associated gene for PCs, suggesting its potentially pivotal role in the underlying mechanisms contributing to the impact of tumor-infiltrating PCs in LUAD.

To further validate the role of TNFRSF17 in LUAD prognosis, we performed immunohistochemical staining to assess the impact of TNFRSF17 in LUAD prognosis in a previous follow-up cohort (Fig. 1H). The results showed that the expression of TNFRSF17 exhibited a tendency to be related to the early pathological stage (Table S7). Additionally, we evaluated disease-free survival and overall survival in the high and low PC groups. The results showed no statistically significant difference in disease-free survival between the two groups (*P* = 0.310), but the high-expression group exhibited better overall survival (*P* = 0.011) (Fig. 1H), consistent with the findings from previous bioinformatics analyses.

Previous studies have yielded conflicting results regarding the impact of tumor-infiltrating PCs on LUAD prognosis. PCs might be one of the primary producers of the immunosuppressive cytokine IL-35 within the stroma, acting as an independent negative prognostic factor for LUAD.^3^ Another study discovered a correlation between higher levels of PCs and extended survival in LUAD.^4^ Our research provides strong evidence supporting the favorable impact of PC infiltration on LUAD prognosis. Moreover, some studies have indicated that PCs play a significant role in tumor immunity.^5^ Hence, we speculate that PCs likely play a crucial regulatory role in the immune response of LUAD, potentially influencing patient prognoses. This hypothesis is supported by our findings using CellChat, highlighting the substantial involvement of PCs in the clinical responses of cancer patients to immune checkpoint inhibitors, challenging the traditional view of their secondary role in anti-tumor responses. Moreover, our study suggests that TNFRSF17 may play a critical role in the potential mechanisms for PC's anti-tumor immunity. This study provides valuable insights into the complex interactions in the tumor microenvironment as well as potential targets for improved LUAD therapy.

## Ethics declaration

All specimens were collected with the informed consent of the patients. This study was approved by the Ethics Committee of the Fourth Affiliated Hospital of China Medical University (Approval No. EC-2023-KS-020).

## Conflict of interests

All authors declare that they do not have competing interests.

## Funding

This work was supported by the support for High-quality Development of Scientific Research Funding Project of China Medical University (No. 2023JH2/20200137).

## Data availability

The scRNA-seq data were obtained from the Gene Expression Omnibus (GEO) at GSE123904 and GSE131907. Bulk RNA-seq and clinical datasets were downloaded from The Cancer Genome Atlas (TCGA) database using the GDC data portal (https://portal.gdc.cancer.gov/) (accessed October 13, 2022).

## Figures and Tables

**figs1:**
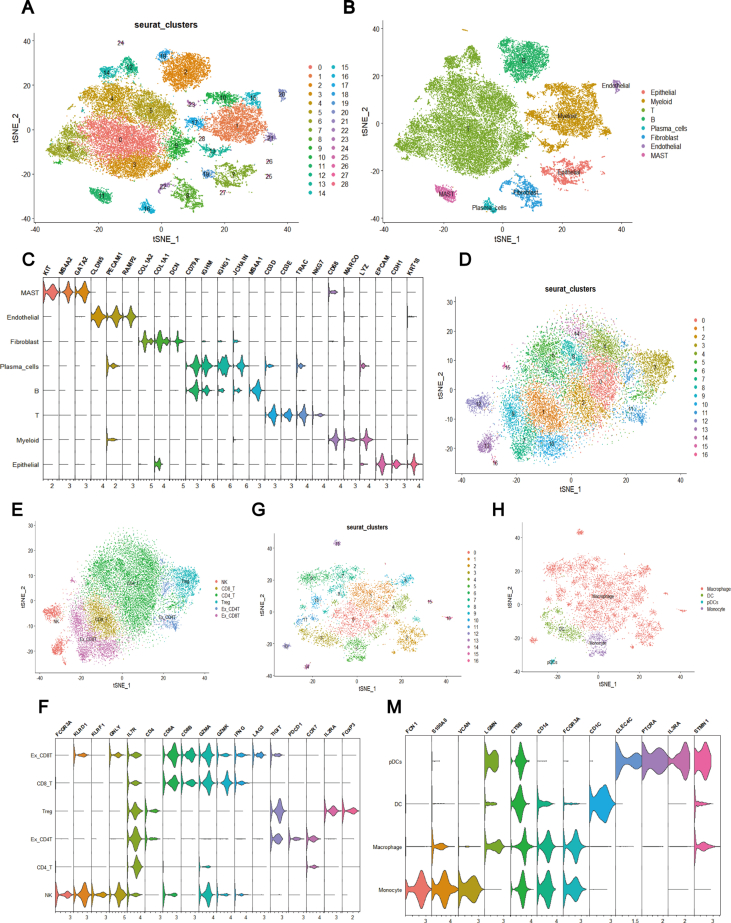


**figs2:**