# Supplementary material for: The important role and core marker gene of tumor-infiltrating plasma cells in the microenvironment of lung adenocarcinoma
Source: Genes Dis. 2024 Mar 22;12(2):101274. doi: 10.1016/j.gendis.2024.101274 (PMC11605347; doi:10.1016/j.gendis.2024.101274)
Supplement: Multimedia component 1 [file mmc1.docx]

**Materials and Methods**

**ScRNA-seq data sources and preprocessing**

We retrieved scRNA-seq sequencing data for LUAD from the Gene Expression Omnibus (GEO) database (http://www.ncbi.nlm.nih.gov/geo/). Specifically, we downloaded scRNA-seq data from GSE123904,^13^ which included four primary LUAD samples, labeled P1T (GSM3516665), P2T (GSM3516672), P3T (GSM3516667), and P4T (GSM3516663). Additionally, we obtained scRNA-seq data from GSE131907,^14^ which comprised four primary LUAD samples labeled P5T (GSM3827125), P6T (GSM3827126), P7T (GSM3827127), and P8T (GSM3827128). These eight samples were subjected to preprocessing steps for further analysis.

Initially, cells with gene expression below 200 or above 7000 were filtered out, as well as cells with mitochondrial content exceeding 20%. Subsequently, the scRNA-seq data were normalized using the 'NormalizeData' function from the 'Seurat' R package. The normalized scRNA-seq data were then transformed into a Seurat object. The 'FindVariableFeatures' function was employed to identify the top 2000 highly variable genes. The Harmony integration algorithm was utilized to integrate the scRNA-seq datasets of the eight samples, yielding a total of 30,323 cells.

**The processing and analysis of scRNA-seq data**

The processing and analysis of scRNA-seq data were performed using the R package. Initially, the 'ScaleData' function was applied to normalize the scRNA-seq data, followed by the analysis of variable features for each sample. The 'Seurat' R package was employed for unsupervised dimensionality reduction and clustering analysis of the gene expression matrix. The 'FindClusters' function was used to assign cells from the integrated data into appropriate clusters. Subsequently, the 'RunTSNE' function was utilized to perform t-distributed stochastic neighbor embedding (t-SNE) for dimensionality reduction. The SingleR package was then applied to annotate these subclusters, with manual curation for refinement. Next, we performed additional clustering and annotation specifically for myeloid cells and T/NK cells. The results of further annotation were merged to replace the previous subclusters. The 'FindAllMarkers' function from the 'Seurat' package was used to test the differential gene expression between a given cluster and all other clusters. To identify marker genes for each cluster, the criteria |log2 (fold change) | > 1 and adjusted p value < 0.05 were used."

**Downloading, organizing, and deconvolution analysis of LUAD bulk RNA sequencing data.**

LUAD RNA sequencing and clinical data were downloaded from The Cancer Genome Atlas (TCGA) database using the GDC data portal (https://portal.gdc.cancer.gov/) (accessed October 13, 2022). Bulk RNA-seq data and clinical information for a total of 503 LUAD patients were obtained by data curation and normalization, and samples with missing clinical information were removed for further survival-related gene screening.

To estimate the cellular composition of LUAD from the entire tumor gene expression data, we employed the cell type deconvolution method based on LUAD-specific gene expression profiles. Specifically, we utilized the "BisqueRNA" (version 1.0.5) method to perform deconvolution on LUAD bulk RNA-seq data obtained from TCGA, enabling us to determine the proportions of different cell types within each sample.^15^ The Bisque reference-based decomposition model relies on extensive RNA-seq count data and a reference dataset that includes read counts from single-cell RNA-seq. Moreover, the single-cell data should be labeled with quantifiable cell types. A reference curve is generated by averaging read count abundances for each cell type in the single-cell data. Given the observed reference spectra and cell proportions in single-cell data, this method learns gene-specific transformations of bulk data to elucidate technical biases between sequencing technologies. Subsequently, Bisque can estimate cell proportions from bulk RNA-seq data using the reference and utilize non-negative least squares (NNLS) regression to estimate cell proportions from transformed bulk expression data.

The estimated proportions within each sample were normalized to a sum of 1 across the included cell types. Differentially expressed genes (DEGs) were identified based on the TCGA Bulk RNA-seq atlas using a threshold of FDR < 0.05 and |log2FC| > 1.

**Pathway and functional enrichment analysis.**

Gene set enrichment analysis was used to identify key pathways and core genes in tumor development.^16^ We selected genes with P < 0.01 and log2FC > 1.5 for gene ontology (GO) and Kyoto Encyclopedia of Genes and Genomes (KEGG) analysis using the R package "clusterProfiler".^17^ KEGG analysis was performed using the enrichKEGG function of the R package "clusterProfiler".^18^ p value < 0.05 was considered significant enrichment.

**CellChat analysis**

CellChat utilizes a comprehensive database of signaling molecule interactions to analyze and visualize intercellular communication in single-cell RNA sequencing (scRNA-seq) data. It considers various aspects of ligand-receptor interactions, including multimeric complexes and different types of signaling molecules. Using mass action models, it infers cell state-specific signal transduction in scRNA-seq data and performs differential expression analyses and statistical tests on cell groups, which can be discrete or continuous on cellular trajectories. Moreover, CellChat offers visual outputs for user-guided data exploration and employs social network analysis tools to quantitatively characterize inferred cell-to-cell communication. This analysis identifies specific signaling roles of cell populations and generalizable rules governing intercellular communication in complex tissues.^19^

**Survival analysis and screening of plasma cell-associated core genes**

After obtaining the composition of cells in each sample through deconvolution, patients were divided into high and low infiltration groups based on the median content of the target cells in each sample based on deconvolution. The risk ratio (HR) for each cell type was calculated using the prognostic data from bulk RNA-seq. Kaplan‒Meier survival curve analysis was performed to estimate the median survival time of the two groups, and the "log-rank" test was conducted to assess the significant difference in survival time between the two groups.

We screened the core genes by focusing on marker genes of plasma cells. First, univariate Cox regression was performed to assess the prognostic value of plasma cell marker genes on overall survival (OS) in TCGA LUAD patients. Subsequently, the random survival forest was employed to identify the core prognostic genes among plasma cell markers.^20^

Meanwhile, DEGs between tumor and normal tissues were screened by limma package among plasma cell marker genes.^21^ The PPI links of DEGs were then analyzed by the STRING (Search Tool for the Retrieval of Interacting Genes) database to screen the more important differential genes. Finally, the key genes for prognosis were found by taking the intersection of differential genes and prognostic genes.

**Collection of clinical specimens and patient data**

A total of 112 paraffin-embedded specimens were collected from patients with LUAD admitted to the Department of Thoracic Surgery at the Fourth Affiliated Hospital of China Medical University from January 1, 2016, to December 31, 2019. None of the patients received radiotherapy, chemotherapy, or immunotherapy prior to surgery. The median follow-up time was 97.5 months (range: 11.13-84.63 months). The following results and clinical data of the 112 patients participating were obtained through medical records review and telephone interviews in this study. The collected data included demographic characteristics (age, gender, smoking and drinking habits), tumor stage (lymph node metastasis, TNM staging), and pathological differentiation. Disease-free survival (DFS) and overall survival (OS) were calculated based on the follow-up records.

**Immunohistochemistry (IHC)**

The expression levels of CD138 and TNFRSF17 were determined by IHC. Envision and DAB staining kits (Antibody Diagnostic Inc., USA) were used for the IHC analysis. In brief, paraffin-embedded specimens were deparaffinized in xylene and a series of gradient ethanol concentrations (100%, 95%, 85%, and 75%). Antigen retrieval was performed using citrate buffer. Subsequently, the specimens were incubated with 10% goat serum at room temperature for 30 minutes. The primary antibodies CD138 (Proteintech, 10593-1-AP-50, USA) diluted at 1:800 and TNFRSF17 (Proteintech, 27724-1-AP50, USA) diluted at 1:150 were applied to the sections and refrigerated overnight at 4℃. The next day, after incubation with secondary antibodies at room temperature for 1 hour, the sections were washed with DAB solution (China Bridge Company) for 10 minutes. Counterstaining was performed using hematoxylin and ammonia water with 1% hydrochloric acid for 20 seconds. The staining results were observed under an optical microscope.

The IHC results were evaluated by two pathologists. CD138 and TNFRSF17 were localized on the cell membrane. We randomly selected five 1-square millimeter regions within the tumor area of the tumor samples and manually counted the positive cells in the selected regions. After calculating the average from all the regions, the average total number of positively stained cells for each marker in each region was expressed as cell density (x/mm2).^22, 23^

**Statistical analysis**

Statistical analysis was performed using SPSS 24.0 software (IBM) and GraphPad Prism 7. The Wilcoxon rank-sum test was used to compare variables between groups. The Cox proportional hazards regression model was employed to calculate independent prognostic factors. p < 0.05 was considered statistically significant.
