# Supplementary material for: The important role and core marker gene of tumor-infiltrating plasma cells in the microenvironment of lung adenocarcinoma
Source: Genes Dis. 2024 Mar 22;12(2):101274. doi: 10.1016/j.gendis.2024.101274 (PMC11605347; doi:10.1016/j.gendis.2024.101274)
Supplement: Multimedia component 2 [file mmc2.docx]

**Processing Workflow for Single-Cell Data**

We obtained and curated sequencing data from eight lung adenocarcinoma patients across two datasets. After applying stringent quality control measures to preprocess the scRNA-seq data, we employed the Harmony algorithm to perform batch correction on the merged scRNA-seq data of the eight patients, resulting in a total of 30,323 cells available for subsequent analysis.

Principal component analysis (PCA) was conducted on the top 2000 variable genes to reduce dimensionality. Using t-SNE analysis and unsupervised clustering (resolution=1), we identified 29 distinct cell clusters (Fig. S1A). The "doubletFinder" R package was utilized to identify doublet cells, which were subsequently excluded from analysis. Furthermore, annotation of these subgroups was performed using the SingleR R package and referenced against a compilation of previously reported marker genes (Table S1) from published literature. The annotated clusters were characterized as B cells, plasma cells, endothelial cells, mast cells, T cells, epithelial cells, dendritic cells, plasmacytoid dendritic cells (pDCs), myeloid cells, and fibroblasts (Fig. S1B). The expression profiles of annotated marker genes within cell clusters were visualized using stacked violin plots (Fig. S1C).

Currently, various functionally distinct subgroups have been identified within myeloid cells and T cells, exerting influence on the occurrence, progression, and prognosis of LUAD. Therefore, to construct a more refined single-cell atlas of LUAD, we extended our annotation approach to myeloid cells and T cells. First, T cells were isolated and subjected to clustering with a resolution of 1.5, resulting in a total of 17 T-cell subclusters (Fig. S1D). Employing the established annotation methodology, we identified six T-cell subtypes, including natural killer (NK) cells, CD8+ T cells, CD4+ T cells, regulatory T cells (Tregs), exhausted CD8+ T cells (EX_CD8T), and exhausted CD4+ T cells (EX_CD4T) (Fig. S1E). The expression profiles of annotated marker genes within T-cell subtypes were visualized through violin plots (Fig. S1F).

Similarly, myeloid cells were clustered with a resolution of 1.5, yielding 17 subclusters (Fig. S1G). Utilizing the previous annotation strategy, we characterized four myeloid cell subtypes, encompassing macrophages, dendritic cells (DCs), plasmacytoid predendritic cells (pDCs), and monocytes (Fig. S1H). The expression patterns of annotated marker genes within myeloid cell subclusters were illustrated using stacked violin plots (Fig. S1M). Subsequently, the further annotated cell subtypes replaced the previous classifications of myeloid cells and T cells, resulting in a total of 16 distinct cell types. Furthermore, using a threshold of logFC > 1 and adj_Pval < 0.05, we identified significantly expressed marker genes within each group.
