# Supplementary material for: The important role and core marker gene of tumor-infiltrating plasma cells in the microenvironment of lung adenocarcinoma
Source: Genes Dis. 2024 Mar 22;12(2):101274. doi: 10.1016/j.gendis.2024.101274 (PMC11605347; doi:10.1016/j.gendis.2024.101274)
Supplement: Multimedia component 3 [file mmc3.docx]

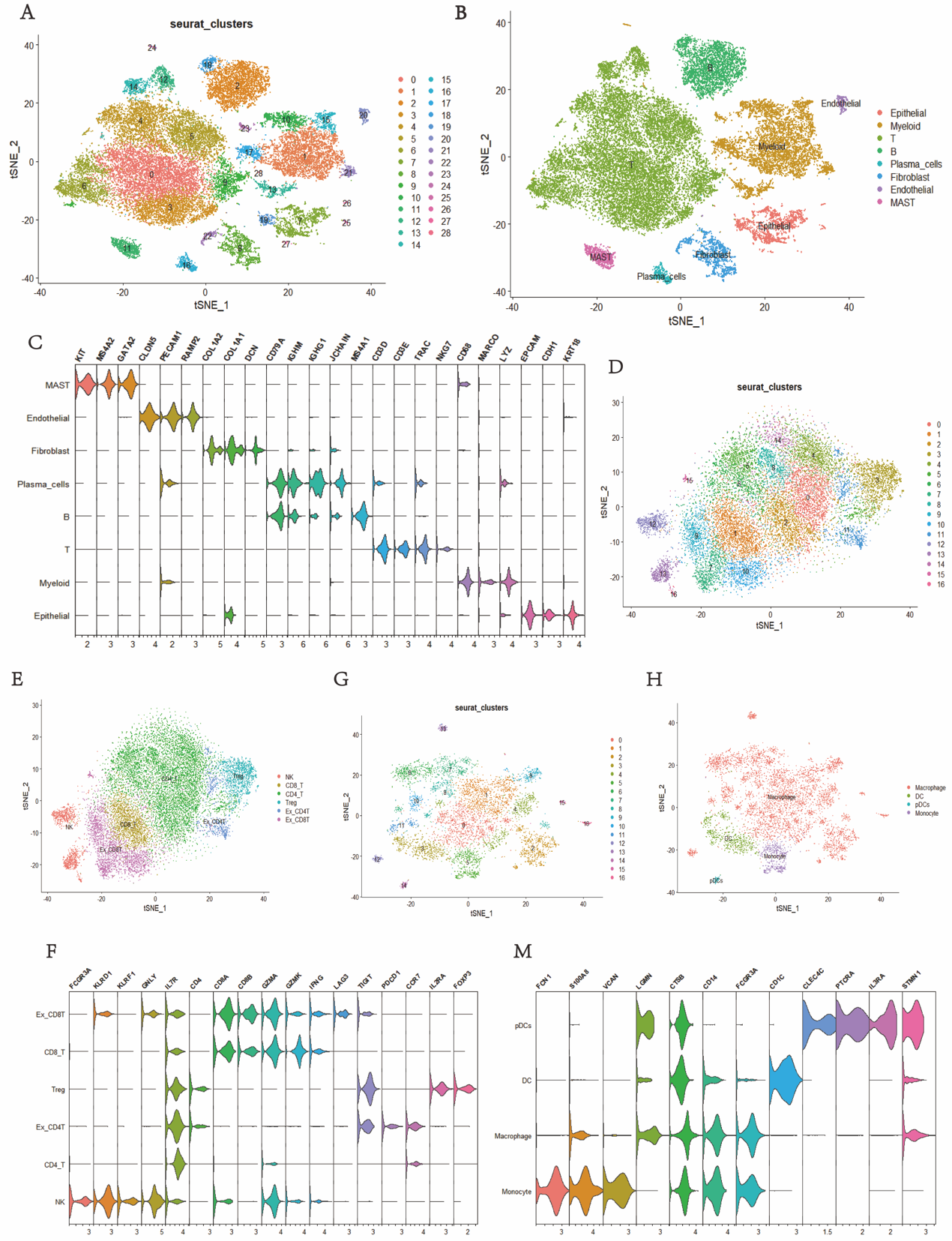


Figure S1: Processing Workflow for Single-Cell Data. (A) A total of 30,323 cells were clustered into 39 clusters. (B) The annotated t-SNE map based on singleR and marker genes. (C) Stacked violin plots showing the expression of annotated marker genes across cell populations. (D) T cells were clustered into 17 cell clusters. (E) t-SNE map of T cells identified as 6 cell subtypes. (F) Stacked violin plots of T-cell marker gene expression. (G) Myeloid cells were clustered into 17 cell clusters. (H) t-SNE map of myeloid cells identified as 4 cell subtypes. (M) Stacked violin plots of myeloid cell marker gene expression.
