# Supplementary material for: The important role and core marker gene of tumor-infiltrating plasma cells in the microenvironment of lung adenocarcinoma
Source: Genes Dis. 2024 Mar 22;12(2):101274. doi: 10.1016/j.gendis.2024.101274 (PMC11605347; doi:10.1016/j.gendis.2024.101274)
Supplement: Multimedia component 4 [file mmc4.docx]

**
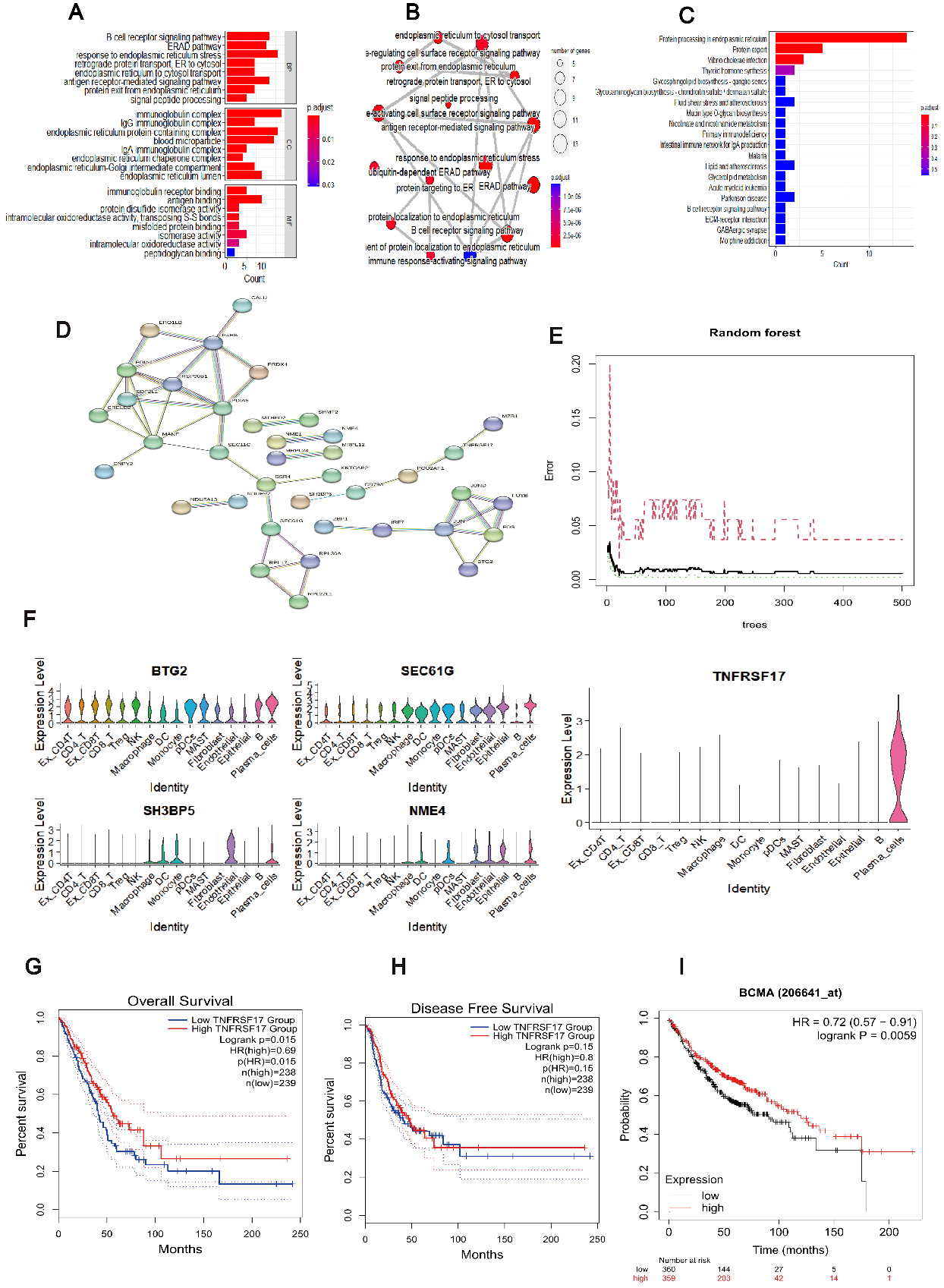
 Figure S2:** **Enrichment analysis of plasma cell marker gene, CellChat analysis and the role of TNFRSF17 in LUAD prognosis.**

(A) GO enrichment analysis.

(B) Interconnections among enriched GO pathways.

(C) KEGG enrichment analysis.

(D) 135 DEGs from 942 PCs marker genes were used for the PPI network using the STRING database, selected with an integrated score > 0.8, and genes without any connections to other nodes were discarded.

(E) Random forest analysis was applied to 117 prognosis-associated genes, 70 genes with a relative importance greater than 0.4 were identified.

(F) Expression of five prognostic genes in PCs was analyzed by scRNA-seq, and TNFRSF17 exhibited specific expression in tumor-infiltrating PCs.

(G) Gepia database online analysis of TNFRSF17 was significantly associated with better survival in LUAD.

(H) Gepia database online analysis of TNFRSF17 was not statistically significant with DFS of LUAD.

(I) The Kaplan‒Meier plotter database showed that TNFRSF17 was significantly associated with better survival in LUAD.
