# Supplementary material for: The important role and core marker gene of tumor-infiltrating plasma cells in the microenvironment of lung adenocarcinoma
Source: Genes Dis. 2024 Mar 22;12(2):101274. doi: 10.1016/j.gendis.2024.101274 (PMC11605347; doi:10.1016/j.gendis.2024.101274)
Supplement: Multimedia component 8 [file mmc8.docx]

Table S4. The clinical relevance of CD138 based on the curated TCGA data

| Characteristics | Low expression of CD138 (n=252) | High expression of CD138 (n=251) | P value |
| --- | --- | --- | --- |
| Age, n (%) |  |  | 0.755 |
| <= 65 | 121 (24.1%) | 117 (23.3%) |  |
| > 65 | 125 (24.9%) | 130 (25.8%) |  |
| Unknown | 6 (1.2%) | 4 (0.8%) |  |
| Gender, n (%) |  |  | 0.505 |
| Female | 140 (27.8%) | 132 (26.2%) |  |
| Male | 112 (22.3%) | 119 (23.7%) |  |
| Pathologic stage, n (%) |  |  | 0.017 |
| Stage I | 124 (24.7%) | 146 (29.0%) |  |
| Stage II | 57 (11.3%) | 63 (12.5%) |  |
| Stage III | 48 (9.5%) | 32 (6.4%) |  |
| Stage IV | 19 (3.8%) | 6 (1.2%) |  |
| Unknown | 4 (0.8%) | 4 (0.8%) |  |
| Tissue or organ of origin, n (%) |  |  | 0.090 |
| Lower lobe | 90 (17.9%) | 82 (16.3%) |  |
| Middle lobe | 13 (2.6%) | 8 (1.6%) |  |
| Upper lobe | 163 (27.0%) | 156 (31.0%) |  |
| Others | 13 (2.6%) | 5 (1.0%) |  |
| Number pack years smoked, n (%) |  |  | 0.745 |
| < 40 | 84 (16.7%) | 83 (16.5%) |  |
| >= 40 | 84 (16.7%) | 91 (18.1%) |  |
| Unknown | 84 (16.7%) | 77 (15.3%) |  |
| Status, n (%) |  |  | 0.068 |
| Alive | 151 (30.0%) | 170 (33.8%) |  |
| Death | 101 (20.1%) | 81 (16.1%) |  |
| Follow-up time (years) | 2.386747 (year) | 2.611090 (year) | 0.306 |
