# Supplementary material for: The important role and core marker gene of tumor-infiltrating plasma cells in the microenvironment of lung adenocarcinoma
Source: Genes Dis. 2024 Mar 22;12(2):101274. doi: 10.1016/j.gendis.2024.101274 (PMC11605347; doi:10.1016/j.gendis.2024.101274)
Supplement: Multimedia component 10 [file mmc10.docx]

Table S6 Association between TNFRSF17 expression and different clinicopathological parameters based on TCGA data.

| Characteristics | Low expression of TNFRSF17 (n=252) | High expression of TNFRSF17 (n=251) | P value |
| --- | --- | --- | --- |
| Age, n (%) |  |  | 0.008 |
| <= 65 | 134 (26.6%) | 104 (20.7%) |  |
| > 65 | 111 (22.1%) | 144 (28.6%) |  |
| Unknown | 7 (1.4%) | 3 (0.6%) |  |
| Gender, n (%) |  |  | 0.262 |
| Female | 130 (25.8%) | 142 (28.2%) |  |
| Male | 122 (24.3%) | 109 (21.7%) |  |
| Pathologic stage, n (%) |  |  | 0.002 |
| Stage I | 69 (13.7%) | 99 (19.7%) |  |
| Stage II | 140 (27.8%) | 129 (25.6%) |  |
| Stage III | 28 (5.6%) | 17 (3.4%) |  |
| Stage IV | 12 (2.4%) | 6 (1.2%) |  |
| Unknown | 3 (0.6%) | 5 (1.0%) |  |
| Tissue or organ of origin, n (%) |  |  | 0.404 |
| Lower lobe | 87 (17.3%) | 85 (16.9%) |  |
| Middle lobe | 14 (2.8%) | 7 (1.4%) |  |
| Upper lobe | 141 (28.0%) | 151 (30.0%) |  |
| Others | 10 (2.0%) | 8 (1.6%) |  |
| Number pack years smoked, n (%) |  |  | 0.681 |
| < 40 | 88 (17.5%) | 79 (15.7%) |  |
| >= 40 | 84 (16.7%) | 91 (18.1%) |  |
| Unknown | 80 (15.9%) | 81 (16.1%) |  |
| Status, n (%) |  |  | 0.206 |
| Alive | 154 (30.6%) | 167 (33.2%) |  |
| Death | 98 (19.5%) | 84 (16.7%) |  |
| Follow-up time (years) | 2.507 | 2.490 | 0.936 |
