# Supplementary material for: The important role and core marker gene of tumor-infiltrating plasma cells in the microenvironment of lung adenocarcinoma
Source: Genes Dis. 2024 Mar 22;12(2):101274. doi: 10.1016/j.gendis.2024.101274 (PMC11605347; doi:10.1016/j.gendis.2024.101274)
Supplement: Multimedia component 11 [file mmc11.docx]

| Characteristics | Low expression of TNFRSF17 (n=56) | High expression of TNFRSF17 (n=56) | P value |
| --- | --- | --- | --- |
| Age, n (%) |  |  | 0.699 |
| <= 65 | 23 (20.5%) | 21 (18.8%) |  |
| > 65 | 33 (29.5%) | 35 (31.3%) |  |
| Gender, n (%) |  |  | 0.701 |
| Female | 34 (30.4%) | 32 (28.6%) |  |
| Male | 22 (19.6%) | 24 (21.4%) |  |
| Pathologic stage, n (%) |  |  | 0.100 |
| Stage I | 35 (31.3%) | 43 (38.4%) |  |
| Stage II+III | 21 (18.8%) | 13 (11.6%) |  |
| Smoking history, n (%) |  |  | 0.563 |
| No | 32 (28.6%) | 35 (31.3%) |  |
| Yes | 24 (21.4%) | 21 (18.8%) |  |
| Drinking History |  |  | 1.000 |
| No | 46 (41.1%) | 46 (41.1%) |  |
| Yes | 10 (8.9%) | 10 (8.9%) |  |
| Recurrence |  |  | 0.844 |
| No | 35 (31.3%) | 36 (32.1%) |  |
| Yes | 21 (18.8%) | 20 (17.9%) |  |
| Status, n (%) |  |  | 0.088 |
| Alive | 37 (33.0%) | 45 (40.2%) |  |
| Death | 19 (17.0%) | 11 (9.8%) |  |
| Follow-up time(year) | 3.450 | 4.593 | 0.959 |

Table S7 Association between TNFRSF17 expression and different clinicopathological parameters.
